# Quality of life from cytoreductive surgery in advanced ovarian cancer: Investigating the association between disease burden and surgical complexity in the international, prospective, SOCQER‐2 cohort study

**DOI:** 10.1111/1471-0528.17041

**Published:** 2022-01-10

**Authors:** Sudha Sundar, Carole Cummins, Satyam Kumar, Joanna Long, Vivek Arora, Janos Balega, Tim Broadhead, Tim Duncan, Richard Edmondson, Christina Fotopoulou, Ros Glasspool, Desiree Kolomainen, Simon Leeson, Ranjit Manchanda, Orla McNally, Jo Morrison, Asima Mukhopadhyay, Jim Paul, John Tidy, Nick Wood

**Affiliations:** ^1^ Institute of Cancer and Genomic Sciences University of Birmingham Birmingham UK; ^2^ Sandwell and West Birmingham NHS Trust Birmingham UK; ^3^ Institute of Applied Health Research University of Birmingham Birmingham UK; ^4^ Bondi Women's Health Sydney NSW Australia; ^5^ Leeds Teaching Hospitals NHS Trust Leeds UK; ^6^ Norfolk & Norwich University Hospital Norwich UK; ^7^ University of Manchester Manchester UK; ^8^ Imperial College London London UK; ^9^ University of Glasgow Glasgow UK; ^10^ Kings College NHS Foundation Trust London UK; ^11^ Betsi Cadwaladr University Health Board Bangor UK; ^12^ Wolfson Institute of Population Health Queen Mary University of London London UK; ^13^ Department of Health Services Research London School of Hygiene & Tropical Medicine London UK; ^14^ Peter MacCallum Cancer Centre Melbourne Vic. Australia; ^15^ Taunton and Somerset NHS Foundation Trust Taunton UK; ^16^ Tata Medical Center Kolkata India; ^17^ University of Sheffield Sheffield UK; ^18^ Lancashire Teaching Hospitals NHS Foundation Trust Preston UK

**Keywords:** extensive surgery, ovarian cancer, quality of life, ultraradical surgery

## Abstract

**Objective:**

To investigate quality of life (QoL) and association with surgical complexity and disease burden after surgical resection for advanced ovarian cancer in centres with variation in surgical approach.

**Design:**

Prospective multicentre observational study.

**Setting:**

Gynaecological cancer surgery centres in the UK, Kolkata, India, and Melbourne, Australia.

**Sample:**

Patients undergoing surgical resection (with low, intermediate or high surgical complexity score, SCS) for late‐stage ovarian cancer.

**Main Outcome Measures:**

Primary: change in global score on the European Organisation for Research and Treatment of Cancer (EORTC) core quality‐of‐life questionnaire (QLQ‐C30). Secondary: EORTC ovarian cancer module (OV28), progression‐free survival.

**Results:**

Patients’ preoperative disease burden and SCS varied between centres, confirming differences in surgical ethos. QoL response rates were 90% up to 18 months. Mean change from the pre‐surgical baseline in the EORTC QLQ‐C30 was 3.4 (SD 1.8, *n* = 88) in the low, 4.0 (SD 2.1, *n* = 55) in the intermediate and 4.3 (SD 2.1, *n* = 52) in the high‐SCS group after 6 weeks (*p* = 0.048), and 4.3 (SD 2.1, *n* = 51), 5.1 (SD 2.2, *n* = 41) and 5.1 (SD 2.2, *n* = 35), respectively, after 12 months (*p* = 0.133). In a repeated‐measures model, there were no clinically or statistically meaningful differences in EORTC QLQ‐C30 global scores between the three SCS groups (*p* = 0.840), but there was a small statistically significant improvement in all groups over time (*p* < 0.001). The high‐SCS group experienced small to moderate decreases in physical (*p* = 0.004), role (*p* = 0.016) and emotional (*p* = 0.001) function at 6 weeks post‐surgery, which resolved by 6–12 months.

**Conclusions:**

The global QoL of patients undergoing low‐, intermediate‐ and high‐SCS surgery improved at 12 months after surgery and was no worse in patients undergoing extensive surgery.

**Tweetable Abstract:**

Compared with surgery of lower complexity, extensive surgery does not result in poorer quality of life in patients with advanced ovarian cancer.

## Tweetable Abstract

Compared with surgery of lower complexity, extensive surgery does not result in poorer quality of life in patients with advanced ovarian cancer.

## 1 INTRODUCTION

Management of advanced ovarian cancer (stages III and IV) comprises cytoreductive surgery and systemic treatment.^1^, ^2^, ^3^ Multiple studies have shown improved progression‐free survival (PFS) and overall survival (OS) where complete macroscopic cytoreduction has achieved no visible residual disease after resection.^4^ Extensive surgery with a high surgical complexity score (SCS) uses procedures such as diaphragm resection and splenectomy to achieve complete macroscopic cytoreduction in patients with higher tumour burden, in an effort to improve their survival.^5^, ^6^, ^7^, ^8^, ^9^ Nevertheless, preoperative disease burden remains a significant prognostic indicator for survival even after achieving complete cytoreduction.^10^ Evidence on outcomes of extensive surgery derives from case series: no randomised controlled trial directly comparing outcomes from extensive surgery versus surgery of low or intermediate complexity for the same preoperative disease burden has been conducted.^11^, ^12^ Meta‐analysis of studies has shown survival benefit from maximal cytoreduction,^13^ but the first population‐level study investigating the impact of the systematic introduction of extensive surgery within a well‐defined algorithm of care showed no overall survival benefit, despite doubling the complete cytoreduction rate.^14^

Both OS and PFS are critical outcomes, but quality of life (QoL) is important to patients in making treatment decisions.^15^, ^16^ Surgical morbidity from extensive surgery is higher,^17^, ^18^ but comparative evidence on the QoL associated with extensive surgery is lacking.^19^ Although the European Organisation for Research and Treatment of Cancer (EORTC) 55971, CHORUS, SCORPION and LION trials have published QoL outcomes, their results do not report on QoL associated with surgery of varying complexity for similar disease burden.^20^

Understanding QoL after extensive surgery for ovarian cancer is critical given three factors: the absence of randomised controlled trial data comparing extensive surgery versus lower complexity surgery for similar disease burden; the clinical challenge of the robust estimation of survival benefit for any individual patient; and the concern that putative survival gain from extensive surgery could be offset by decreased QoL from increased morbidity.^21^, ^22^

A single‐centre pilot study found that QoL after high‐SCS procedures for higher disease burden declined postoperatively, but recovered within 9 months to levels comparable with that experienced by patients undergoing low‐ or intermediate‐SCS procedures.^23^ The SOCQER‐2 study investigated QoL following extensive (high‐SCS or ‘ultra‐radical’) surgery compared with low‐ or intermediate‐SCS surgery in a prospective observational multicentre study design. The a priori hypothesis, based on the pilot study finding, was that QoL in patients undergoing high‐SCS surgery would reduce in the short term postoperatively but would recover to levels comparable with that of patients undergoing less complex surgery by 12 months after surgery.^24^ SOCQER‐2 was commissioned by the UK National Institute for Health and Care Excellence (NICE) in order to inform future guidance for ovarian surgery in the UK. The study is reported following Strengthening the Reporting of Observational studies in Epidemiology (STROBE) criteria.

## 2 METHODS

### 2.1 Study design and patient cohorts

SOCQER‐2 was a prospective, non‐randomised observational study run as parallel studies across the UK, India and Australia. Participating centres aimed to identify and recruit consecutive participants prior to surgical treatment. The recruitment period was from September 2015 to September 2016, with follow‐up until disease progression or death over 24 months.

Patients were eligible if they had suspected or confirmed epithelial ovarian cancer with radiological spread beyond pelvis and if primary (PDS) or delayed debulking surgery (DDS) was planned. Patients receiving neoadjuvant chemotherapy could be recruited prior to chemotherapy or immediately prior to DDS. Patients who did not have International Federation of Gynecology and Obstetrics (FIGO) stage‐III or ‐IV epithelial ovarian cancer on histology following surgery, or who did not undergo debulking surgery as planned, were subsequently excluded.

Data collected at baseline included Eastern Cooperative Oncology Group (ECOG) Performance Status^25^ and the modified age‐adjusted Charlson comorbidity index (ACCI).^26^, ^27^ Disease burden was assessed by peritoneal carcinomatosis index (PCI) pre‐ and post‐surgery, and intraoperative disease mapping (IOM) was used to identify the highest level of abdominal disease.^28^, ^29^ Surgical data collection captured details of the surgeries performed and any intra‐ and postoperative complications up to 6 weeks, which were coded using the Clavien–Dindo classification.^30^ The validated Aletti SCS was used define surgical complexity: low (score 1–3), intermediate (score 4–7) or high (score 8+).^31^, ^32^, ^33^ Pancreatic tail resection, cholecystectomy, resection from lesser sac and porta hepatis disease were not included in the original score and were allocated a score of 5: this score modification did not alter the SCS grouping of patients. Data were recorded using the REDcap platform on a secure server.^34^

### 2.2 Quality‐of‐life measures

Patients completed the validated patient‐reported outcome measure (PROM) questionnaires EORTC QLQ‐C30 and EORTC QLQ‐OV28 at baseline or before surgery for patients undergoing neoadjuvant chemotherapy,^35^ ^,^ ^36^ and then postoperatively at 6 weeks and at 6, 12, 18 and 24 months.^37^, ^38^ Patients were offered a choice of postal or online data collection using the secure QTool system.^39^ Questionnaire completion ceased upon disease progression. The translation of EORTC QLQ‐OV28 into Bengali was performed in line with EORTC guidelines.^40^, ^41^ A change in score of 5–10 points on the EORTC QLC‐C30 global scale was considered small, a change of 10–20 points was considered moderate and a change of 20+ points was considered large.^15^ A change of 10 points was considered clinically meaningful, in line with EORTC 55971.^42^ We also described the direction of change in the EORTC QLQ‐C30 global scale.^15^

### 2.3 Eligibility/selection of centres

To ensure that patients undergoing procedures with a range of surgical complexity were included, high‐ and medium‐volume gynaecological cancer centres self‐declared their practice prior to study participation: some had incorporated high‐SCS procedures, where appropriate given the patient’s disease, into routine practice, to varying degrees; others had not. UK gynaecological cancer centres conform to standards set by the Royal College of Obstetricians and Gynaecologists (RCOG) and are staffed by trained subspecialists in gynaecological oncology. Centres in Kolkata, India, and Melbourne, Australia, were staffed by gynaecological oncologists trained in the UK.

### 2.4 Outcome measures

The primary outcome measure was change in EORTC QLQ‐C30 global score following surgical treatment, measured at 6 weeks, 6 months and 12 months after surgery; secondary outcomes were EORTC QLQ‐C30 dimensional and functional scores and EORTC OV28 score at 6 weeks, 6 months and 12 months after surgery, and PFS and OS at 2 years. A complete case general linear repeated‐measures analysis of variance comparing SCS groups was performed, using change from the pre‐surgery baseline EORTC QLQ‐C30 global score at 6 weeks, 6 months and 12 months post‐surgery, with the baseline score fitted as a covariate. Tests for sphericity and fit were carried out. Post hoc comparisons were made using Bonferroni’s adjustment. Outcomes were analysed by SCS groups, regardless of whether patients underwent PDS or DDS: this decision was based on trials showing QoL as being equivalent in these groups.^20^ Further models, however, included: PDS versus DDS; maximum level of disease; and SCS, PDS versus DDS and maximum level of disease. Data were not considered to be missing at random and there was no data imputation. In line with our hypothesis that differences in QoL between groups would be maximal at 6 weeks and resolved by 12 months, we also compared mean change scores at those time points using all available data. Analysis of subscale outcomes was considered exploratory.

Kaplan–Meier survival analysis and Cox proportional hazard regression using a forward stepwise procedure were carried out for PFS and OS at 2 years. Progression was as defined by the treating clinician. Variables included in the Cox proportional hazard models were SCS (low, intermediate or high), baseline treatment plan (DDS or PDS), pre‐surgical albumin level of <35 g/L or ≥35 g/L, aged ≥65 or <65 years, ACCI of <2 or ≥2, highest level of disease and preoperative PCI (<5, 6–14 or ≥15), with likelihood ratio tests of contribution to model determining inclusion and exclusion in the models at each step. All statistical analysis was conducted in spss 24 (IBM, Armonk, NY, USA).

### 2.5 Sample size calculation

A sample size calculation was used to identify the minimum number needed to detect a clinically meaningful difference in QOL between intermediate/low‐SCS and high‐SCS surgery. Assuming that the ratio of group sizes for high‐SCS surgery to intermediate‐SCS surgery was 2:1, α = 0.05, a power of 80%, a 13‐point difference in EORTC QLC‐30 of clinical importance and a baseline score of 66 (SD 24) in those undergoing high‐SCS surgery,^41^ a sample size of 123 (intermediate = 41 and extensive = 82) would be required, with an additional allowance for dropout (calculations were performed in stata 13.1; StataCorp, College Station, TX, USA). This was the minimum recruitment target to satisfy the commissioning organisation’s requirements, but recruitment was planned to continue until the end of the 1‐year period to maximise the statistical power with consideration of confounding factors.

## 3 RESULTS

### 3.1 Demographics of recruited cohort

**FIGURE 1:**
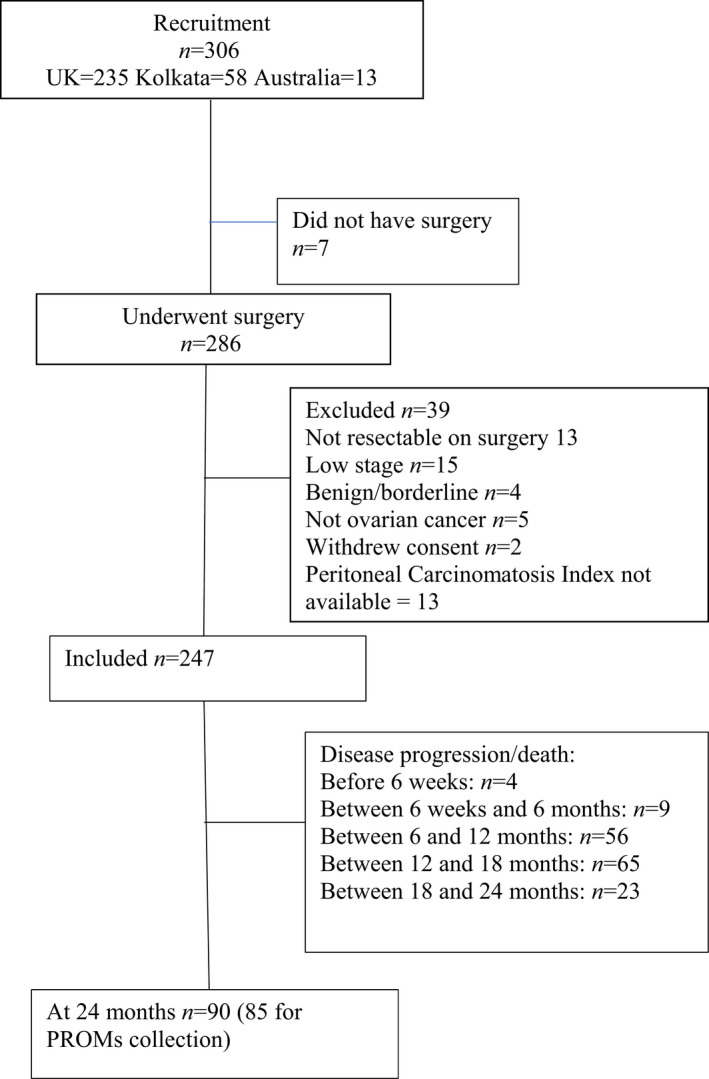
Study flow diagram

A total of 293 patients were recruited from 12 cancer centres in the UK (*n* = 235) and one centre in India (*n* = 58) over a period of 12 months. After surgery and histopathology, 247 (84%) were eligible for inclusion (Figure 1). Cancer registration data for England indicates that English centres recruited 25% of women with late‐stage ovarian cancer presenting for surgical resection in the whole recruitment period within their surgical catchment areas, with a range of 10–57% at different centres: this range reflects the staggered set‐up of the centres and, in some cases, research nurse vacancies. The centre in Australia recruited 13 patients (12 with low‐SCS surgery and one with intermediate‐SCS surgery), but the PCI scores were not available and so those patients were not considered in the analysis of QoL, as adjustment for disease burden was not possible. More patients in the intermediate‐ and high‐SCS groups were <65 years old, with better performance status and lower comorbidity measured by the ACCI (Table 1).

**TABLE 1:** Baseline and postoperative patient characteristics by modified Aletti surgical complexity score (SCS) group

Patient characteristics	Low SCS *N* = 113		Intermediate SCS *N* = 70		High SCS *N* = 64		*p*
	Number	%	Number	%	Number	%	
Age in years							
≤65 years	51	45.1	44	62.9	48	75	0.001
>65 years	62	54.9	26	37.1	16	25	
ECOG performance status							
0	53	46.9	35	50	19	29.7	0.046
1	52	46	25	35.7	36	56.3	
2, 3, 4	8	7.1	10	14.3	9	14.1	
Age‐adjusted Charlson comorbidity index							
0–2	62	54.9	49	70	46	71.9	0.033
3+	51	45.1	21	30	18	28.1	
Body mass index (kg/m^2^)							
≤25	42	37.2	37	52.9	31	48.4	0.096
>25	69	61.1	32	45.7	33	51.6	
Timing of surgery							
PDS	10	8.8	26	37.1	39	60.9	0.001
NACT	103	91.2	44	62.9	25	39.1	
Pre‐surgery haemoglobin							
≤109 g/L	49	43.4	28	40.0	25	39.1	0.827
>110 g/L	64	56.6	42	60.0	39	60.9	
Pre‐surgery albumin level							
>35 g/L	22	19.5	14	20	17	26.6	0.511
>5 g/L	91	80.5	56	80	47	73.4	
Peritoneal Carcinomatosis Index							
≤6	65	57.5	18	25.7	2	3.1	0.001
7–12	21	18.6	29	41.4	6	9.4	
>12	27	23.9	23	32.9	56	87.5	
Level/distribution of disease							
Level 1 (highest level of disease: pelvis)	20	17.7	7	10	0	0	0.001
Level 2 (highest level of disease: mid‐abdomen)	45	39.8	19	27.1	5	7.8	
Level 3 (highest level of disease: upper abdomen)	48	42.5	44	62.9	59	92.2	
Outcome of surgery: residual disease							
None visible	63	55.8	50	71.4	40	62.5	0.007
≤1 cm	29	25.7	17	24.3	21	32.8	
>1 cm	21	18.6	3	4.3	3	4.7	
Final FIGO stage							
3A/3B	11	9.7	9	12.9	2	3.1	0.068
3C	68	60.2	34	48.6	33	51.6	
4	31	27.4	26	37.1	29	45.3	
Postoperative chemotherapy							
Carboplatin (C) ± taxol (T)	106	94	62	89	62	97	0.591
C + T + bevacizumab	20	18	15	21	8	13	
Other	5	4	5	7	2	3	
No chemotherapy	2	2	3	4	0	0	
UK/India patient							
UK (*n* = 195)	108	95.6	53	75.7	34	53.1	0.001
India (*n* = 52)	5	4.4	17	24.3	30	46.9	
Pre‐surgery EORTC QLQ‐C30 global score, mean (SD)	65.1 (21.7)		59.8 (19.9)		58.1 (22.2)		0.094
	**Median, days**	**IQR**	**Median, days**	**IQR**	**Median, days**	**IQR**	
Length of hospital admission	5	3	6	3	9	8	0.001
Surgery to chemotherapy interval	31	16	31	13	39	20	0.005

DDS, delayed debulking surgery; NACT, neoadjuvant chemotherapy; PDS, primary debulking surgery.

### 3.2 Characterisation of disease burden in patient cohort

The preoperative median PCI was 11 (IQR 13) and 85/247 (34%) had a PCI of ≤6, 56/247 (23%) had a PCI of 7–12 and 106/247 had a PCI >12. Low‐, intermediate‐ and high‐SCS procedures were performed in 46% (113), 28% (70) and 26% (64) of patients, respectively. Upper abdominal disease was present in 43% (48), 63% (44) and 92% (59) of patients undergoing low‐, intermediate‐ or high‐SCS procedures, respectively (*p* = 0.001) (Table 1). Patients undergoing low‐SCS procedures had PCI and level‐of‐disease scores that overlapped with those undergoing intermediate procedures, but patients undergoing high‐SCS procedures had a higher disease burden, as defined by a higher PCI score and more extensive upper abdominal disease (*p* = 0.001) (Figure S1; Table 1).

In the 70% (187) of patients undergoing DDS, 103 (60%) had low‐, 44 (25%) had intermediate‐ and 25 (15%) had high‐SCS surgery. Among the 30% (75) undergoing PDS, 10 (13%) patients had low‐, 26 (35%) had intermediate‐ and 39 (52%) had high‐SCS surgery (*p* = 0.001) (Table 1). Both the patients’ preoperative PCI and the complexity of surgery varied across participating centres (Figure S1), reflecting differences in surgical ethos (*p* = 0.001) (Table 1). Preoperative PCI was lower in women undergoing DDS than in women undergoing PDS (data not shown).

### 3.3 Quality of life

Response rates for patients undergoing intermediate‐ or high‐SCS surgery were >80% of those eligible across all time points, but were lower for patients undergoing low‐SCS surgery, with 70% responding at 12–18 months and 46% responding at 24 months (Table S1). A minority chose electronic data collection, with many of these changing to postal data collection over the course of the study.

**FIGURE 2:**
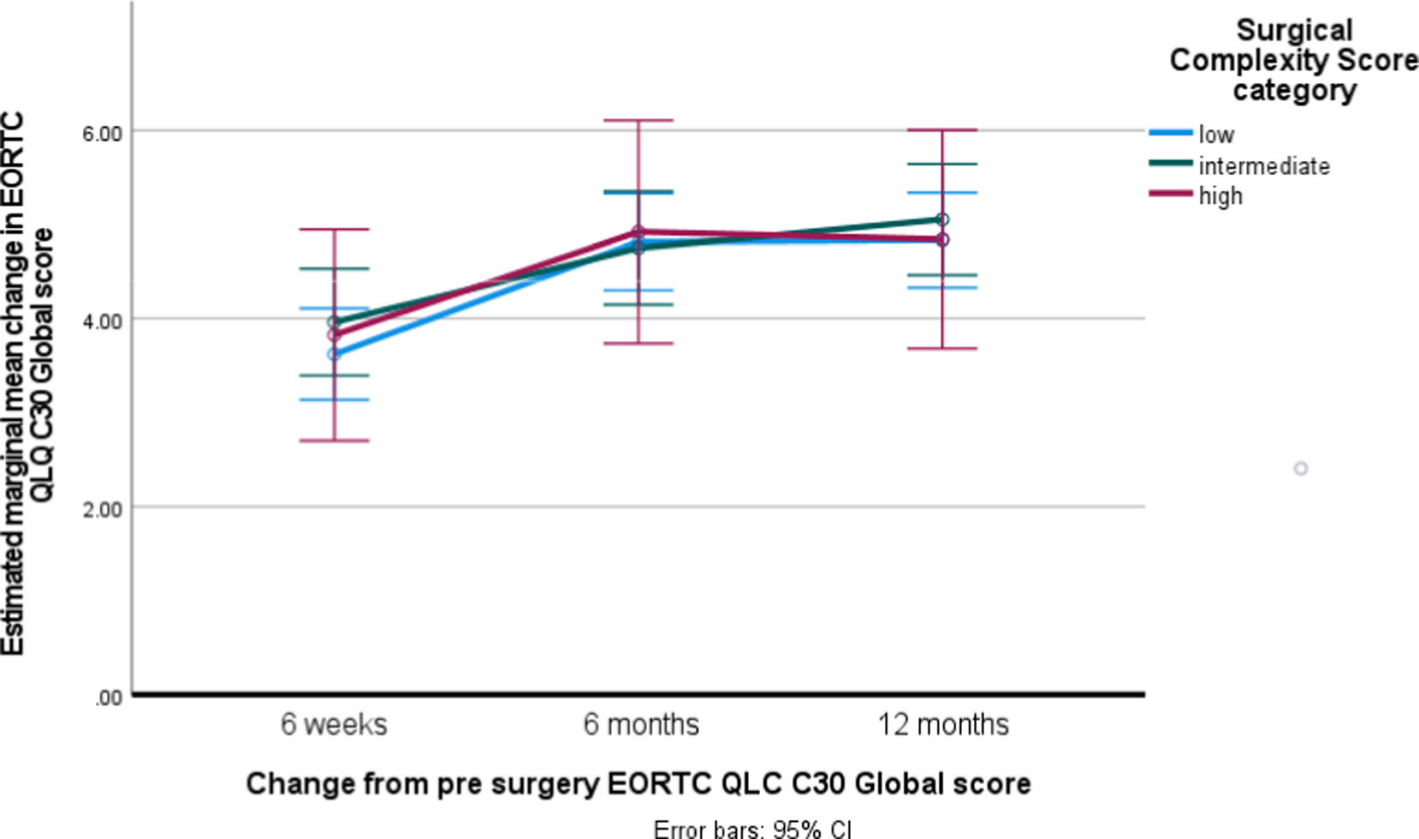
Change in EORTC QLQ‐C30 global score from surgical baseline, by surgical complexity

The mean change in score from the pre‐surgical baseline in the EORTC QLQ‐C30 at 6 weeks post‐surgery was 3.4 (SD 1.8, *n* = 88) in the low‐SCS group, 4.0 (SD 2.1, *n* = 55) in the intermediate‐SCS group and 4.3 (SD 2.1, *n* = 52) in the high‐SCS group (*p* = 0.048). At 12 months post‐surgery the mean change was 4.3 (SD 2.1, *n* = 51) in the low‐SCS group, 5.1 (SD 2.2, *n* = 41) in the intermediate‐SCS group and 5.1 (SD 2.2, *n* = 35) in the high‐SCS group (*p* = 0.133) (Table 2). In a complete case repeated‐measures analysis of variance of change from the pre‐surgical baseline EORTC QLQ‐C30 global score at 6 weeks, 6 months and 12 months post‐surgery, with the baseline score fitted as a covariate, there were no clinically or statistically meaningful differences in EORTC QLQ‐C30 global scores between the three SCS groups (*p* = 0.840), but there was a small statistically significant improvement over time in all patients, irrespective of SCS score, QOL showed a small statistically significant improvement post surgery over the 12 months duration. (*p* < 0.001) (Figure 2). Mean scores allowing comparison with EORTC reference values are given in Table S2. In further models PDS versus DDS and maximum level of disease were not associated with changes in the EORTC QLQ‐C30 global score.

**TABLE 2:** Estimated mean change in EORTC QLQ‐C30 global scores by SCS group with pre‐surgery score as a covariate

SCS score	6 weeks post‐surgery			6 months post‐surgery			12 months post‐surgery		
	Estimated mean	95% CI		Estimated mean	95% CI		Estimated mean	95% CI	
Low	−2.9	−8.1	2.3	8.5	2.9	14.1	7.5	1.9	13.2
Intermediate	−1.4	−7.1	4.4	8.9	2.7	15.0	8.4	2.2	14.7
High	−0.1	−6.7	6.5	2.9	−4.1	10.0	7.1	1.0	14.2

The EORTC QLQ‐C30 physical function (*p* = 0.004), role (*p* = 0.001) and emotional function (*p* = 0.016), but not the global score, were lower in the high‐SCS group at 6 weeks post‐surgery, but by 12 months there was no difference in physical and emotional function between the three groups (Table S2). In all groups clinically meaningful and statistically significant improvements in physical function were noted at 12 months post‐surgery. There were no differences between the groups with regards to cognitive or social function, both of which improved over time. Intermediate‐ and high‐SCS groups had higher financial difficulty symptom scores, with no other differences in symptom scales both pre‐ and post‐surgery (Table S3): this may be related to the younger age profile of these SCS groups. There were no differences in EORTC QLQ‐OV28 scores between SCS groups at 12 months post‐surgery (Table S4).

When considering the direction of change in EORTC QLQ‐C30 scores from baseline at 6 weeks post‐surgery: 43 (48.9%) of patients who had undergone low‐SCS surgery, 23 (41.8%) of those who had undergone intermediate‐SCS surgery and 19 (35.9%) of those who had undergone high‐SCS surgery experienced a negative change in EORTC QLQ‐C30 global score, whereas 23 (26.1%), 22 (40%) and 23 (44.2%) patients, respectively, experienced a positive change (*p* = 0.219). At 12 months post‐surgery, 17 (33.1%) of the patients who had undergone low‐SCS surgery, eight (19.5%) of those who had undergone intermediate‐SCS surgery and ten (28.6%) of those who had undergone high‐SCS surgery had a negative change in EORTC QLQ‐C30 global score, whereas 24 (47.1%), 27 (65.9%) and 23 (65.7%) patients, respectively, experienced a positive change (*p* = 0.180) (Table S5).

A total of 15 out of 27 (55.6%) patients with stomas who responded reported a negative change at 6 weeks post‐surgery, one reported no change and eight reported a positive change in EORTC QLQ‐C30 global score, compared with 75/179 (41.2%) with no stoma reporting a negative change and 63 reporting a positive change. One patient subsequently had a loop ileostomy following obstruction during chemotherapy. At 12 months post‐surgery, nine out of 28 (32.1%) patients with stomas reported a negative change, one reported no change and eight reported a positive change in EORTC QLQ‐C30, compared with 27/111 (24.3%) with no stoma reporting a negative change and 67 (60.4%) reporting a positive change. There was no difference in the distribution of the EORTC QLQ‐C30 global score at 6 weeks or at 12 months post‐surgery between those with and without stomas.

Differences in EORTC QLQ‐C30 at 18 and 24 months post‐surgery were measured with less precision, as more of the patients experienced disease progression. At these time points the completion rates of the questionnaire from the low‐SCS group were poorer than from the intermediate‐ and high‐SCS groups, suggesting a biased response (Table S6).

### 3.4 Surgical outcomes

Complete macroscopic tumour clearance was achieved in 56% (63), 71% (50) and 63% (40) of patients undergoing low‐, intermediate‐ or high‐SCS procedures, respectively (*p* = 0.007) (Table 1). More women in the low‐SCS group had residual disease, 50/113 (44%), reflecting the presence of upper abdominal disease in 43% of the low‐SCS group (Table 1).

Liver mobilisation and diaphragmatic peritonectomy or resections were performed in 53 (22%) patients and splenectomy was performed in 21 (9%) patients. Large bowel resection was performed in 60/247 patients, 38 of whom received end colostomy (15%) and 22 of whom received primary anastomoses (9%). In total, 30% of patients sustained at least one minor or major postoperative complication (Table S7). Complication rates varied by SCS type (low SCS, 20%; intermediate SCS, 26%; high SCS, 52%; *p* < 0.001). In all, 14.2% had grade‐3 or higher complications: 9% of the low‐SCS group, 13% of the intermediate‐SCS group and 25% of the high‐SCS group. Three patients died from complications of surgery: a woman undergoing intermediate‐SCS surgery developed disseminated intravascular coagulation and multi‐organ failure; a woman aged 76 years undergoing low‐SCS surgery died as a result of a pulmonary embolism; and a woman undergoing intermediate‐SCS surgery with intraoperative blood loss of 2–3 L developed intra‐abdominal sepsis.

### 3.5 Survival

Cumulative PFS at 2 years was 34% (95% CI 24.7–42.3%) for the low‐SCS group, 47% (95% CI 35.0–58.6%) for the intermediate‐SCS group and 34% (95% CI 22.4–46%) for the high‐SCS group (*p* = 0.109) (Figure S2). In forward stepwise Cox regression models that included level of disease, preoperative PCI, ACCI, residual disease, preoperative albumin level, age, initial treatment strategy (PDS or DDS) and country, only comorbidity as measured by ACCI and upper abdominal disease, and not SCS surgical group, were associated with PFS (Table S8). In patients with only pelvic disease PFS was 57% (95% CI 36.8–74.4%), in those with mid‐abdominal disease PFS was 49% (95% CI 37.4–61.0%) and in those with upper abdominal disease PFS was 29% (95% CI 21.4–36.0%) (*p* = 0.001).

Patients with no residual disease status after surgery had better PFS (47% versus 21%; *p* < 0.001) and OS (83% versus 64%; *p* < 0.001) at 2 years post‐surgery. There were no differences in PFS or OS according to whether patients received PDS or DDS or by their country of residence and treatment (India or UK; data not included).

## 4 DISCUSSION

### 4.1 Main findings

We found that patients with late‐stage ovarian cancer had no important differences in EORTC QLQ‐C30 global scores measured across 6 weeks, 6 months and 12 months post‐surgery when undergoing surgery of varying complexity, despite a higher preoperative disease burden in patients undergoing the most complex surgery. Across all SCS groups, global QoL showed a small but significant improvement by 12 months postoperatively. Patients who underwent the most complex surgery (high‐SCS group) had small to moderate detriments in EORTC QLQ‐C30 physical function, role function and emotional function at 6 weeks post‐surgery compared with patients undergoing less extensive surgery (intermediate‐ and low‐SCS groups), but by 6–12 months post‐surgery these functions are comparable across all SCS categories. A majority of women undergoing high‐SCS surgery without disease progression experienced a positive change in W

QoL by 12 months post‐surgery. Our methodologically robust multicentre study confirms findings from smaller single‐centre studies.^24^, ^43^

Those undergoing high‐SCS procedures had significantly greater disease burden and more upper abdominal disease, but patients with these disease characteristics also underwent surgery of low or intermediate complexity. As some women with comparably high disease burden would not have been offered surgery, understanding the QoL and survival of these patients not undergoing surgery is essential if the true value or detriment from high‐SCS surgery is to be assessed. We hypothesise that, where high‐complexity surgery is not part of routine practice, fewer patients with a high disease burden on imaging preoperatively will be offered surgery. This interpretation is in keeping with the results from the national ovarian cancer audit from England, which demonstrated that only 51% of women with advanced ovarian cancer undergo surgery.^44^

Patients undergoing low‐complexity surgery had higher rates of residual disease and lower survival compared with those with a similar disease burden undergoing surgery of intermediate complexity. These patients, however, were older with higher comorbidity and lower performance status. The presence of upper abdominal disease and pre‐existing comorbidities was associated with poorer PFS and OS. Postoperative residual disease was associated with poorer OS, particularly in patients undergoing low‐complexity surgery.

### 4.2 Strengths

Study strengths include a clear hypothesis and a design that addressed patient and disease confounding factors. This is the first study that has investigated QoL following surgeries of different complexity while accounting for disease burden. Centres with differing surgical approaches participated in the study with careful data collection on disease burden and distribution. Validated QoL instruments were used and the production of a validated Bengali translation for EORTC QLQ‐OV28 ensured that non‐English speaking patients in Kolkata were able to participate, and that as far as possible the QoL assessments were comparable between centres in Kolkata and the UK. There were minimal missing data (>99% data fields complete for clinical and surgical information, 88% PROMs response) and minimal loss to follow‐up in the period up to 12 months post‐surgery.

### 4.3 Limitations

Limitations of the study are the cohort design: randomisation would be the gold standard to evaluate survival and QoL. However, given the lack of equipoise amongst surgeons, with strong belief in the value (or lack of it) of high‐SCS procedures to achieve complete cytoreduction, a clinical trial would be challenging to deliver. We cannot exclude selection bias, but recruitment to this study was carried out by research nurses, and therefore systematic bias introduced by surgeons recruiting patients whom they believed would recover well after extensive surgery is unlikely. Continuing research by the team will use cancer registration data to investigate bias in the choice of patients for surgical intervention by comparing the recruited patients in each centre with the ‘denominator’ total patient cohort in each centre.

We recruited fewer women undergoing high‐complexity surgery and more women undergoing low‐complexity surgery than we expected at the time of sample size calculation, somewhat reducing our anticipated power regarding the outcomes of high‐SCS surgery. There were, however, no population‐based data on the proportion and demographics of patients undergoing high‐complexity procedures from the UK or internationally. A comparative study between two centres in the UK identifies variations in the extent of cytoreductive surgery.^45^ On a larger scale, results from the population‐based national ovarian cancer audit in England has demonstrated significant geographical variation in the rates of surgery.^44^ Similarly, registry data from the Netherlands shows significant variation in the proportion undergoing complete cytoreductive surgery,^46^ whereas in the USA, only 48% of ovarian cancer surgery is guideline compliant.^45^ These papers confirm that the true utilisation of extensive surgery/high‐SCS procedures on a population basis in the ‘real world’, as opposed to that reported in academic publications from selected centres, is simply not known. Furthermore, publications on outcomes from high‐SCS surgery rarely present total cohort ‘denominator’ data.^14^, ^22^

### 4.4 Interpretation in light of other evidence

Studies have shown that maximal‐effort cytoreductive surgery improves survival from advanced ovarian cancer. Evidence on QoL in patients undergoing extensive/high‐complexity surgery compared with surgery of lower complexity for similar disease burden is scarce. Our study shows that QoL improved over 12 months, compared with preoperative scores, for the majority of patients undergoing low/intermediate‐ or high‐SCS procedures. High‐complexity cytoreductive surgery did not result in poorer QoL compared with intermediate‐ or low‐complexity procedures. There were no clinically meaningful differences in QoL among patients undergoing surgery of different complexities.

### 4.5 Recommendation for practice

Patients undergoing high‐complexity surgery can be reassured that by 12 months post‐surgery most will have better QoL after than immediately before surgery.

### 4.6 Research recommendation

Our findings on variation in practice, surgical ethos, distribution of disease burden in surgeries of different complexity and outcomes are novel but highly likely to be generalisable across health systems. Research is needed to understand the reasons for this variation in surgical approach, its relationship with survival outcomes and algorithms that can improve the standardisation of surgical decision making.

## 5 CONCLUSION

There can be confidence in clinical practice that the use of high‐complexity surgery in advanced ovarian cancer will not have a significant or clinically meaningful detrimental effect on global QoL compared with less complex surgery. Short‐term impacts on physical function, emotional and role domains need to be discussed with patients and appropriate support provided to women undergoing extensive surgery.

## CONFLICT OF INTEREST

SS has received honoraria from Astra Zeneca, MSD and GSK outside the submitted work. CF has received honoraria from Ethicon, Tesaro, MSD/Astra Zeneca, Clovis, Roche, GSK. RM reports grants from Barts Charity, grants from The Eve Appeal, personal fees from Astra Zeneca, MSD, outside the submitted work. RE reports personal fees from Astra Zeneca, personal fees from Clovis Pharma, personal fees from GSK, outside the submitted work. Completed disclosure of interests form available to view online as supporting information. AM reports royalty from Newcastle University (Clovis Oncology) related to the work of development of rucaparib. This is unrelated to the submitted work.

## AUTHOR CONTRIBUTIONS

SS and CC secured funding, designed and conducted the study. SK, JL conducted the study, collected the data and SK, JL and CC analysed results from the study. All co‐authors contributed intellectually to the design of the study, contributed clinical data and interpreted the results of the study for clinical practice. All authors reviewed the manuscript prior to submission. Authorship order for all authors apart from the study team at Birmingham is based in alphabetical order.

## ETHICAL APPROVAL

Ethical approval was obtained (UK, ref. no. 15/WM/0124; India, ref. no. EC/TMC/68/16; Australia ref. no. HREC/15/MH/126).

## Supporting information

Supplementary Material

Supplementary Material

Supplementary Material

Supplementary Material

Supplementary Material

Supplementary Material

Supplementary Material

Supplementary Material

Supplementary Material

Supplementary Material

Supplementary Material

Supplementary Material

Supplementary Material

Supplementary Material

Supplementary Material

Supplementary Material

Supplementary Material

Supplementary Material

Supplementary Material

## Data Availability

The data that support the findings of this study are available on request from the corresponding author. The data are not publicly available due to privacy or ethical restrictions.
