# Supplementary material for: Quality of life from cytoreductive surgery in advanced ovarian cancer: Investigating the association between disease burden and surgical complexity in the international, prospective, SOCQER‐2 cohort study
Source: BJOG. 2022 Jan 10;129(7):1122–32. doi: 10.1111/1471-0528.17041 (PMC9306902; doi:10.1111/1471-0528.17041)
Supplement: Supplementary file 1 — Supplementary Material [file BJO-129-1122-s009.docx]

**Figure S1: Distribution of Peritoneal carcinomatosis by Surgical complexity score type amongst recruited patients in study**

**S1B: Distribution of pre-operative PCI by SCS group**


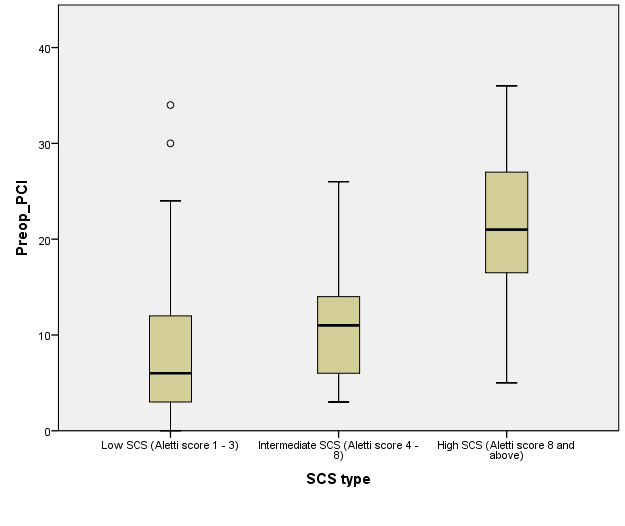


**S1B: Distribution of pre-operative PCI by participating centres**


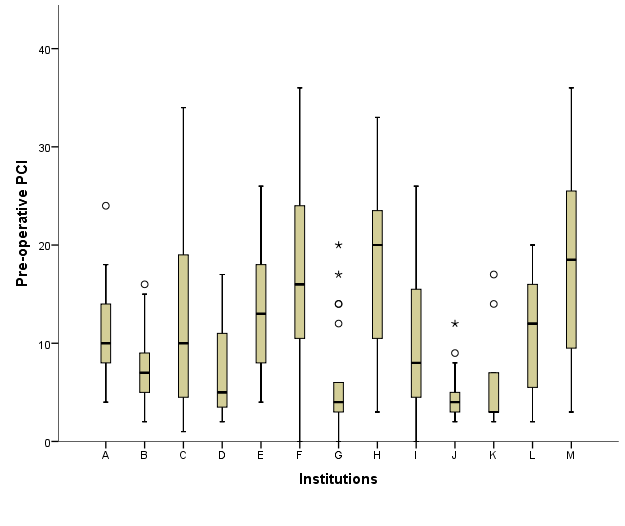


**Figure S2A: Cumulative progression free survival by SCS type up to 2 years**


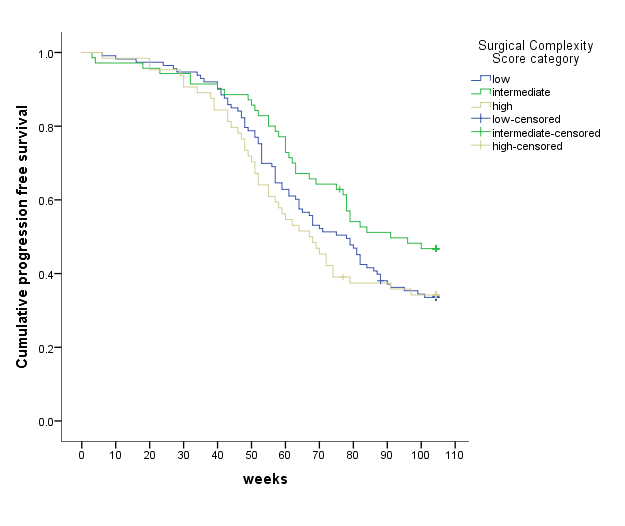


**S 2B: Cumulative overall survival by SCS type up to 2 years**


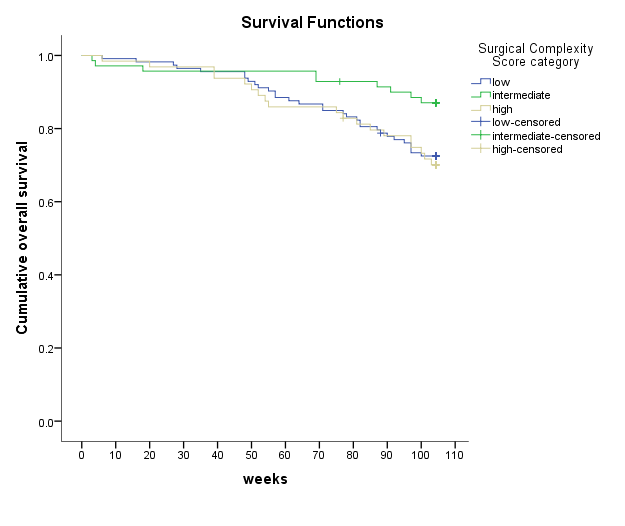


**Table S1: Patient reported outcome measures (PROMs) completion rate and loss to follow-up**

| Time points | PROMs data completed | PROMs data expected (n=247) | Percentage of PROMs data completed | Reasons for change in eligible participants at each time point – cumulative numbers |
| --- | --- | --- | --- | --- |
| Baseline | 221 | 242 | 91.3% | 5 withdrew consent for PROMs data collection |
| 6 weeks | 217 | 238 | 91.2% | 1 progressed / 3 deaths |
| 6 months | 205 | 229 | 89.5% | 9 progressed |
| 12 months | 142 | 173 | 82.1% | 56 progressed |
| 18 months | 103 | 108 | 95.4% | 65 progressed |
| 24 months | 61 | 85 | 71.2% | 23 progressed |

**Table S2: EORTC QLQ-C30 in patients by Surgical Complexity Score group**

| EORTC QLQ C30 | Types of surgery | Pre-surgery | | | 6 weeks | | | 6 months | | | 12 months | | | p value* |
| --- | --- | --- | --- | --- | --- | --- | --- | --- | --- | --- | --- | --- | --- | --- |
|  |  | N | Mean | SD | N | Mean | SD | N | Mean | SD | N | Mean | SD |  |
| Global QoL | Low | 98 | 65.1 | 21.7 | 98 | 59.9 | 19.5 | 93 | 69.9 | 19.8 | 57 | 72.2 | 20.5 | **<0.001 |
|  | Intermediate SCS | 62 | 59.8 | 19.9 | 61 | 60.1 | 19.3 | 53 | 67.3 | 21.5 | 44 | 74.4 | 18.6 | ***0.539 |
|  | High | 59 | 58.1 | 22.2 | 56 | 60.1 | 18.9 | 52 | 66.5 | 20.1 | 38 | 73.5 | 16.9 |  |
|  | p |  | | | 0.986# | | |  | | | 0.867# | | |  |
| Functional QoL: Physical function | Low | 99 | 75.7 | 19.8 | 97 | 64.5 | 20.2 | 93 | 76.3 | 21.5 | 56 | 80.6 | 18.9 | **<0.00 |
|  | Intermediate SCS | 60 | 74.0 | 23.1 | 59 | 67.8 | 18.0 | 54 | 73.2 | 17.6 | 45 | 77.9 | 20.8 | ***0.009 |
|  | High SCS | 60 | 73.4 | 20.9 | 50 | 55.6 | 18.6 | 54 | 64.4 | 24.2 | 40 | 76.5 | 19.3 |  |
|  | P |  | | | 0.004# | | | 0.007# | | | 0.528# | | |  |
| Functional QoL: Role function | Low | 99 | 66.5 | 29.0 | 99 | 45.1 | 27.5 | 94 | 70.7 | 27.4 | 57 | 80.1 | 25.1 | **<0.001 |
|  | Intermediate SCS | 61 | 65.8 | 28.8 | 60 | 52.8 | 26.8 | 55 | 67.9 | 29.7 | 45 | 75.2 | 24.8 | ***0.070 |
|  | High | 59 | 64.7 | 27.9 | 57 | 39.2 | 23.9 | 56 | 60.7 | 33.7 | 40 | 69.6 | 29.5 |  |
|  | p |  | | | 0.016# | | |  | | | 0.166# | | |  |
| Functional QoL: Emotional function | Low | 98 | 78.1 | 21.1 | 98 | 76.8 | 21.4 | 93 | 79.7 | 20.6 | 56 | 78.6 | 22.2 | **0.430 |
|  | Intermediateee SCS | 62 | 63.7 | 27.2 | 61 | 72.4 | 20.8 | 55 | 75.8 | 19.7 | 44 | 75.4 | 19.0 | ***0.005 |
|  | High | 59 | 62.6 | 25.4 | 57 | 69.3 | 19.4 | 55 | 69.2 | 25.7 | 39 | 73.3 | 23.4 |  |
|  | p |  | | | 0.034# | | | 0.036# | | | 0.548# | | |  |
| Functional QoL: Cognitive function | Low | 99 | 78.3 | 21.2 | 99 | 75.9 | 21.1 | 91 | 78.2 | 21.3 | 56 | 81.3 | 20.1 | **0.732 |
|  | Intermediate SCS | 62 | 76.3 | 26.2 | 61 | 78.1 | 21.2 | 55 | 78.2 | 20.8 | 45 | 79.3 | 24.9 | ***0.731 |
|  | High | 60 | 76.7 | 24.0 | 57 | 76.0 | 19. | 56 | 76.5 | 25.0 | 40 | 81.7 | 22.6 |  |
|  | p |  | | | 0.672# | | |  | | | 0.820# | | |  |
| Functional QoL: Social function | Low | 98 | 65.1 | 29.4 | 98 | 58.8 | 28.1 | 93 | 74.9 | 26.1 | 56 | 84.5 | 22.2 | **<0.00  **1 |
|  | Intermediate SCS | 61 | 58.7 | 29.9 | 59 | 60.5 | 26.2 | 55 | 71.5 | 31.5 | 45 | 83.0 | 21.8 | **0.213 |
|  | High | 60 | 63.6 | 29.2 | 57 | 57.0 | 29.9 | 56 | 64.6 | 30.3 | 39 | 79.5 | 23.1 |  |
|  | p |  | | | 0.850# | | |  | | | 0.481# | | |  |

*complete case general linear repeated measures (one way ANOVA) at 12 months ** within group change across 6 weeks, 6 months and 12 months

*** between SCS group change

# Kruskal-Wallis test all available data at that time point

**Table S3: EORTC QLQ-C30: Symptoms scales by Surgical Complexity Score group**

| Symptom scale | Types of surgery | Pre-surgery | | | 6 weeks | | | 6 months | | | 12 months | | | p value |
| --- | --- | --- | --- | --- | --- | --- | --- | --- | --- | --- | --- | --- | --- | --- |
|  |  | N | Mean | SD | N | Mean | SD | N | Mean | SD | N | Mean | SD |  |
| Fatigue | Low SCS | 96 | 38.3 | 22.9 | 99 | 51.1 | 23.5 | 93 | 33.1 | 24.7 | 57 | 28.9 | 22.2 | *<0.001 |
|  | Intermediate SCS | 61 | 38.1 | 22.2 | 61 | 42.4 | 21.8 | 55 | 39.6 | 24.2 | 45 | 30.6 | 22.2 | **0.798 |
|  | High SCS | 60 | 36.5 | 21.5 | 57 | 49.1 | 21.3 | 56 | 40.7 | 27.8 | 40 | 26.7 | 20.9 |  |
|  | p |  | | | 0.064# | | |  | | | 0.682# | | |  |
| Nausea | Low SCS | 99 | 8.2 | 19.4 | 98 | 14.1 | 21.2 | 93 | 7.5 | 16.8 | 57 | 8.2 | 16.4 | *0.030 |
|  | Intermediate SCS | 59 | 14.7 | 24.4 | 60 | 13.9 | 19.2 | 55 | 11.5 | 23.1 | 45 | 10.7 | 21.4 | **0.336 |
|  | High SCS | 59 | 11.6 | 15.2 | 57 | 15.5 | 21.3 | 56 | 11.6 | 23.1 | 40 | 7.5 | 13.6 |  |
|  | p |  | | | 0.997# | | |  | | | 0.897# | | |  |
| Pain | Low SCS | 99 | 19.9 | 24.0 | 98 | 32.1 | 26.7 | 93 | 19.2 | 23.8 | 57 | 16.4 | 19.0 | *<0.001 |
|  | Intermediate SCS | 61 | 26.2 | 27.1 | 61 | 28.1 | 24.4 | 55 | 28.5 | 27.0 | 45 | 22.2 | 26.1 | **0.772 |
|  | High SCS | 60 | 23.9 | 23.6 | 57 | 29.8 | 22.7 | 56 | 26.5 | 28.4 | 40 | 14.2 | 18.3 |  |
|  | p |  | | | 0.600# | | |  | | | 0.371# | | |  |
| Dyspnoea | Low SCS | 99 | 21.5 | 25.8 | 99 | 19.5 | 25.2 | 94 | 22.3 | 26.5 | 56 | 14.3 | 21.9 | *0.468 |
|  | Intermediate SCS | 61 | 15.8 | 25.5 | 61 | 16.4 | 23.3 | 54 | 22.8 | 30.9 | 45 | 13.3 | 25.0 | **0.837 |
|  | High SCS | 60 | 17.8 | 20.8 | 57 | 18.1 | 27.5 | 56 | 17.9 | 23.8 | 40 | 15.8 | 21.3 |  |
|  | p |  | | | 0.679# | | |  | | | 0.611# | | |  |
| Insomnia | Low SCS | 100 | 33.3 | 32.5 | 98 | 40.8 | 33.7 | 94 | 30.5 | 30.8 | 56 | 30.4 | 33.2 | *0.007 |
|  | Intermediate SCS | 61 | 36.1 | 31.8 | 58 | 39.7 | 33.9 | 54 | 35.2 | 31.3 | 45 | 27.4 | 27.8 | **0.812 |
|  | High SCS | 60 | 35.6 | 32.4 | 57 | 36.3 | 31.7 | 56 | 32.7 | 32.7 | 40 | 25.8 | 29.7 |  |
|  | p |  | | | 0.752# | | |  | | | 0.843# | | |  |
| Appetite | Low SCS | 100 | 20.3 | 27.6 | 99 | 33.0 | 29.5 | 94 | 15.6 | 25.7 | 57 | 15.8 | 25.3 | *<0.001 |
|  | Intermediate SCS | 61 | 31.1 | 32.7 | 61 | 25.7 | 28.8 | 55 | 13.9 | 24.6 | 45 | 10.4 | 22.3 | **0.208 |
|  | High SCS | 60 | 31.7 | 31.5 | 57 | 33.9 | 32.4 | 56 | 23.8 | 31.6 | 40 | 7.5 | 19.2 |  |
|  | p |  | | | 0.240# | | |  | | | 0.093# | | |  |
| Constipation | Low SCS | 100 | 20.3 | 26.8 | 99 | 37.0 | 34.6 | 94 | 15.6 | 24.3 | 56 | 20.8 | 28.8 | *<0.001 |
|  | Intermediate SCS | 61 | 24.0 | 29.3 | 61 | 37.7 | 33.6 | 55 | 21.8 | 30.2 | 45 | 16.3 | 25.2 | **0.556 |
|  | High SCS | 59 | 26.0 | 31.6 | 57 | 35.7 | 33.8 | 56 | 23.2 | 33.6 | 40 | 17.5 | 26.1 |  |
|  | p |  | | | 0.947# | | |  | | | 0.716# | | |  |
| Diarrhoea | Low SCS | 98 | 10.5 | 22.2 | 98 | 10.2 | 18.2 | 94 | 7.8 | 17.2 | 57 | 8.8 | 18.4 | *0.079 |
|  | Intermediate SCS | 62 | 11.3 | 23.3 | 61 | 13.1 | 23.0 | 55 | 12.1 | 23.5 | 45 | 7.4 | 15.7 | **0.204 |
|  | High SCS | 59 | 10.2 | 21.7 | 57 | 17.5 | 26.8 | 55 | 12.7 | 20.8 | 40 | 13.3 | 25.9 |  |
|  | p |  | | | 0.292# | | |  | | | 0.818# | | |  |
| Financial difficulty | Low SCS | 98 | 14.3 | 26.2 | 97 | 12.7 | 21.8 | 94 | 13.1 | 24.5 | 56 | 6.5 | 14.8 | *0.103 |
|  | Intermediate SCS | 62 | 31.2 | 35.6 | 60 | 24.4 | 34.1 | 55 | 27.3 | 35.2 | 44 | 18.2 | 26.4 | **0.002 |
|  | High SCS | 59 | 21.5 | 33.8 | 57 | 26.3 | 35.5 | 56 | 21.4 | 32.7 | 40 | 22.5 | 29.6 |  |
|  | p |  | | | 0.062# | | |  | | | 0.005# | | |  |

* within group change across 6 weeks, 6 months and 12 months ** between SCS group change # Kruskal-Wallis test all available data at that time poin**t**

**Table S4: EORTC QLQ-OV28 symptom scales by SCS group**

| EORTC QLQ OV28 | Types of surgery | Pre-surgery | | | 6 weeks | | | 6 months | | | 12 months | | | 18 months | | | 24 months | | | p value |
| --- | --- | --- | --- | --- | --- | --- | --- | --- | --- | --- | --- | --- | --- | --- | --- | --- | --- | --- | --- | --- |
| Symptom scale |  | N | Mean | SD | N | Mean | SD | N | Mean | SD | N | Mean | SD | N | Mean | SD | N | Mean | SD |  |
| Abdominal pain | Low SCS | 96 | 21.5 | 19.1 | 95 | 28.0 | 17.8 | 92 | 18.1 | 17.3 | 92 | 18.1 | 17.3 | 39 | 16.1 | 13.5 | 20 | 17.2 | 14.1 | *<0.001 |
|  | Intermediate. SCS | 62 | 32.7 | 26.3 | 60 | 29.3 | 20.7 | 55 | 24.0 | 22.6 | 55 | 24.0 | 22.6 | 33 | 15.8 | 20.1 | 22 | 14.1 | 17.4 | **0.142 |
|  | High SCS | 59 | 36.6 | 22.6 | 57 | 29.8 | 18.2 | 56 | 23.0 | 15.5 | 55 | 22.6 | 15.4 | 31 | 22.8 | 17.2 | 18 | 23.5 | 21.7 |  |
|  | p |  | | | 0.820# | | |  | | | 0.091# | | |  | | |  | | |  |
| Peripheral neuropathy | Low SCS | 99 | 26.8 | 33.1 | 99 | 32.2 | 35.6 | 94 | 33.9 | 32.8 | 56 | 28.0 | 30.8 | 39 | 22.2 | 27.7 | 19 | 28.9 | 27.7 | *<0.001 |
|  | Intermediate. SCS | 60 | 17.2 | 29.4 | 61 | 25.4 | 32.1 | 53 | 39.6 | 33.2 | 45 | 34.8 | 35.3 | 33 | 28.8 | 26.1 | 22 | 21.2 | 23.1 | **0.837 |
|  | High SCS | 59 | 9.3 | 18.6 | 57 | 14.9 | 20.3 | 56 | 42.0 | 32.9 | 40 | 32.9 | 32.4 | 31 | 31.2 | 29.1 | 19 | 31.6 | 30.9 |  |
|  | p |  | | | 0.021# | | |  | | | 0.610# | | |  | | |  | | |  |
| Hormonal symptoms | Low SCS | 99 | 20.2 | 27.8 | 99 | 24.6 | 28.8 | 94 | 24.3 | 32.5 | 57 | 24.6 | 30.2 | 39 | 23.5 | 27.2 | 20 | 20.0 | 28.4 | *0.067 |
|  | Intermediate. SCS | 62 | 23.9 | 32.9 | 61 | 29.5 | 33.0 | 54 | 30.6 | 33.8 | 44 | 26.9 | 29.2 | 33 | 24.2 | 24.0 | 22 | 18.9 | 20.8 | **0.525 |
|  | High SCS | 59 | 15.8 | 25.8 | 57 | 12.3 | 21.9 | 56 | 17.9 | 29.8 | 40 | 25.8 | 29.9 | 31 | 24.7 | 28.5 | 19 | 11.4 | 24.2 |  |
|  | p |  | | | 0.003# | | |  | | | 0.847# | | |  | | |  | | |  |
| Body image | Low SCS | 97 | 32.6 | 27.1 | 99 | 38.0 | 25.5 | 94 | 32.1 | 25.9 | 57 | 25.7 | 28.7 | 39 | 17.1 | 21.1 | 20 | 18.3 | 25.9 | *0.001 |
|  | Intermediate. SCS | 62 | 34.9 | 24.1 | 59 | 39.8 | 30.5 | 54 | 34.6 | 32.4 | 45 | 32.2 | 28.3 | 33 | 15.7 | 27.3 | 22 | 22.0 | 27.4 | **0.396 |
|  | High SCS | 59 | 32.5 | 25.2 | 57 | 41.8 | 26.9 | 56 | 39.9 | 30.6 | 40 | 24.2 | 27.7 | 31 | 36.6 | 29.9 | 19 | 38.6 | 32.9 |  |
|  | p |  | | | 0.641# | | |  | | | 0.235# | | |  | | |  | | |  |
| Attitude to disease / treatment | Low SCS | 95 | 48.2 | 25.0 | 99 | 52.7 | 25.7 | 93 | 44.6 | 29.6 | 56 | 37.3 | 24.8 | 39 | 29.3 | 24.5 | 20 | 33.3 | 29.5 | *0.001 |
|  | Intermediate. SCS | 60 | 55.4 | 25.3 | 59 | 53.3 | 24.7 | 54 | 49.4 | 30.8 | 45 | 44.7 | 27.6 | 33 | 29.6 | 23.5 | 22 | 31.8 | 23.3 | **0.703 |
|  | High SCS | 60 | 50.7 | 21.1 | 56 | 54.8 | 26.9 | 56 | 48.4 | 28.6 | 39 | 36.2 | 30.1 | 31 | 38.4 | 27.9 | 19 | 52.0 | 26.2 |  |
|  | p |  | | | 0.831# | | |  | | | 0.306# | | |  | | |  | | |  |
| Chemotherapy side effects | Low SCS | 98 | 25.0 | 17.2 | 98 | 27.3 | 17.4 | 92 | 24.7 | 19.3 | 54 | 24.9 | 18.6 | 39 | 23.4 | 18.1 | 20 | 25.3 | 21.5 | *0.660 |
|  | Intermediate. SCS | 61 | 23.4 | 18.0 | 61 | 26.0 | 17.2 | 54 | 27.7 | 17.2 | 43 | 27.3 | 18.7 | 33 | 21.2 | 18.3 | 22 | 14.5 | 13.6 | **0.491 |
|  | High SCS | 59 | 17.9 | 14.9 | 57 | 21.2 | 13.5 | 56 | 23.3 | 13.9 | 39 | 20.9 | 15.2 | 31 | 20.2 | 17.3 | 19 | 17.2 | 19.2 |  |
|  | p |  | | | 0.092# | | |  | | | 0.317# | | |  | | |  | | |  |
| Other symptoms | Low SCS | 71 | 42.4 | 20.5 | 67 | 50.7 | 19.0 | 38 | 41.0 | 23.4 | 15 | 31.1 | 21.7 | 10 | 24.2 | 17.8 | 5 | 20.0 | 15.1 | *0.004 |
|  | Intermediate. SCS | 34 | 44.6 | 26.3 | 37 | 42.8 | 22.4 | 37 | 42.3 | 20.0 | 20 | 30.4 | 25.8 | 19 | 21.1 | 21.4 | 12 | 16.7 | 20.1 | **0.392 |
|  | High SCS | 36 | 32.4 | 21.2 | 29 | 38.2 | 18.3 | 48 | 40.6 | 17.8 | 31 | 23.7 | 18.1 | 26 | 24.0 | 17.8 | 15 | 18.3 | 13.4 |  |
|  | p |  | | | 0.010# | | |  | | | 0.482# | | |  | | |  | | |  |

* within group change across 6 weeks, 6 months and 12 months ** between SCS group change # Kruskal-Wallis test all available data at that time poin**t**

**Table S5: Direction of change in EORTC QLQ-C30 Global score from pre surgery baseline at 6 weeks and 12 monhts post operation**

|  | Surgical Complexity Score | | | | | |
| --- | --- | --- | --- | --- | --- | --- |
| Change in EORTC QLQ-C30 Global score from pre surgery baseline | low |  | intermediate | | high |  |
|  | Count | % | Count | % | Count | % |
| **6 weeks post surgery:** |  |  |  |  |  |  |
| Any negative change | 43 | 48.9 | 23 | 41.8 | 19 | 43.6 |
| *large negative change* | *18* | *20.5* | *9* | *16.4* | *8* | *15.4* |
| *moderate negative change* | *13* | *14.8* | *9* | *16.4* | *7* | *13.5* |
| *a little negative change* | *12* | *13.6* | *5* | *9.1* | *7* | *7.7* |
| No change | 22 | 25.0 | 10 | 18.2 | 10 | 19.2 |
| Any positive change | 23 | 26.1 | 22 | 40.0 | 23 | 44.2 |
| *a little positive change* | *8* | *9.1* | *1* | *1.8* | *1* | *3.8* |
| *moderate positive change* | *11* | *12.5* | *14* | *25.5* | *12* | *19.2* |
| *large positive change* | *4* | *4.5* | *7* | *12.7* | *12* | *21.2* |
| Total | 88 |  | 55 |  | 52 |  |
| **12 months post surgery:** |  |  |  |  |  |  |
| Any negative change | 17 | 33.3 | 8 | 19.5 | 10 | 28.6 |
| *large negative change* | *8* | *15.7* | *5* | *12.2* | *5* | *14.3* |
| *moderate negative change* | *3* | *5.9* | *3* | *7.3* | *1* | *2.9* |
| *a little negative change* | *6* | *11.8* | *0* | *0.0* | *4* | *11.4* |
| No change | 10 | 19.6 | 6 | 14.6 | 2 | 5.7 |
| Any positive change | 24 | 47.1 | 27 | 65.9 | 23 | 65.7 |
| *a little positive change* | *5* | *9.8* | *2* | *4.9* | *1* | *2.9%* |
| *moderate positive change* | *9* | *17.6* | *9* | *22.0* | *8* | *22.9%* |
| *large positive change* | *10* | *19.6* | *16* | *39.0* | *14* | *40.0%* |
| Total | 51 |  | 41 |  | 35 |  |

**Table S6: EORTC QLC-C30 scores at 18 months and 24 months**

| EORTC QLQ-C30 | | 18 months | |  |  | 24 months | |  |  |
| --- | --- | --- | --- | --- | --- | --- | --- | --- | --- |
|  |  | N | Median | IQR | | N | Median | IQR | |
| Global | Low SCS | 39 | 75.00 | 66.67 | 83.33 | 20 | 75 | 58.33 | 83.33 |
|  | Intermediate SCS | 32 | 83.33 | 66.67 | 83.33 | 20 | 83.33 | 66.67 | 87.50 |
|  | High SCS | 29 | 66.67 | 50.00 | 83.33 | 16 | 70.83 | 50.00 | 83.33 |
| Kruskal-Wallis test | | p=0.022 | | | | p=0.416 | | | |
| Physical function* | Low SCS | 39 | 86.67 | 66.67 | 93.33 | 20 | 86.67 | 63.33 | 96.67 |
|  | Intermediate SCS | 32 | 90.00 | 76.67 | 93.33 | 22 | 86.67 | 80.00 | 100.00 |
|  | High SCS | 31 | 80.00 | 66.67 | 93.33 | 19 | 93.33 | 80.00 | 100.00 |
| Role function* | Low SCS | 39 | 100.00 | 66.67 | 100.00 | 20 | 100 | 50.00 | 100 |
|  | Intermediate SCS | 33 | 100.00 | 66.67 | 100.00 | 22 | 100 | 83.33 | 100 |
|  | High SCS | 31 | 66.67 | 50 | 100.00 | 19 | 83.33 | 50.00 | 100 |
| Emotional function* | Low SCS | 39 | 75.00 | 66.67 | 100.00 | 20 | 79.17 | 62.50 | 100 |
|  | Intermediate SCS | 33 | 83.33 | 75.00 | 100.00 | 22 | 83.33 | 75.00 | 100 |
|  | High SCS | 31 | 75.00 | 50.00 | 83.33 | 19 | 75.00 | 50.00 | 91.67 |
| Cognitive function* | Low SCS | 39 | 83.33 | 66.67 | 100.00 | 20 | 83.33 | 66.67 | 91.67 |
|  | Intermediate SCS | 33 | 83.33 | 83.33 | 100.00 | 22 | 83.33 | 83.33 | 100.00 |
|  | High SCS | 31 | 83.33 | 66.67 | 100.00 | 19 | 83.33 | 66.67 | 100.00 |
| Social function* | Low SCS | 39 | 100.00 | 66.67 | 100.00 | 20 | 100 | 75.00 | 100.00 |
|  | Intermediate SCS | 33 | 100.00 | 83.33 | 100.00 | 21 | 100 | 66.67 | 100.00 |
|  | High SCS | 31 | 83.33 | 50.00 | 100.00 | 18 | 58.33 | 50.00 | 100.00 |

* Kruskal Wallis test not statistically significant for all functional scales at 18 and 24 months

**Table S7 : Intra-operative and post operative complications by SCS**

| \|  \| **Intra-operative complications** \| \| \| \| \| \| **Post-operative complications** \| \| \| \| \| \| --- \| --- \| --- \| --- \| --- \| --- \| --- \| --- \| --- \| --- \| --- \| --- \| \| **SCS type** \| **Haemorrhage** \| **Urinary tract injury** \| **GI injury** \| **Vascular injury** \| **Anaesthetic complications** \| **Total, n (%)** \| **C-D class 2, conservative management**  **n (%)** \| **C-D class 3, Radiological/ surgical management without GA**  **n (%)** \| **C-D class 4, ITU/Operative management under GA**  **n (%)** \| **C-D class 5, Death**  **n (%)** \| **Total,**  **n (%)** \| \| Low (n=113) \| 0 \| 2 \| 2 \| 1 \| 2 \| 7 (6.2) \| 12 \| 9 \| 0 \| 1 \| 22 (19.5) \| \| Intermediate (n=70) \| 0 \| 2 \| 1 \| 2 \| 1 \| 6 (8.6) \| 9 \| 6 \| 1 \| 2 \| 18 (25.7) \| \| High (n=64) \| 1 \| 1 \| 2 \| 2 \| 1 \| 7 (10.9) \| 17 \| 9 \| 7 \| 0 \| 33 (51.6) \| \| Total (n=247) \| 1 \| 5 \| 5 \| 5 \| 4 \| 20 (8.1) \| 38 (15.4) \| 24 (9.7) \| 8 (3.2) \| 3 (1.2) \| 73 (29.6) \| |
| --- | --- | --- | --- | --- | --- | --- | --- | --- | --- | --- | --- | --- | --- | --- | --- | --- | --- | --- | --- | --- | --- | --- | --- | --- | --- | --- | --- | --- | --- | --- | --- | --- | --- | --- | --- | --- | --- | --- | --- | --- | --- | --- | --- | --- | --- | --- | --- | --- | --- | --- | --- | --- | --- | --- | --- | --- | --- | --- | --- | --- | --- | --- | --- | --- | --- | --- | --- | --- | --- | --- | --- | --- |

**Table S8: Progression free and overall survival adjusted hazard ratios up to two years**

**a) Progression free survival**

|  | Hazard ratio (Exp(b)) | 95.0% CI | |
| --- | --- | --- | --- |
| ACCI >2 | 1.62 | 1.18 | 2.23 |
| Pelvic disease only and (reference) | 1 |  |  |
| Pelvic and mid abdominal disease | 1.34 | 0.69 | 2.58 |
| Upper abdominal and other disease | 2.34 | 1.29 | 4.26 |

**b) Overall survival**

|  | Hazard ratio (Exp(b)) | 95.0% CI | |
| --- | --- | --- | --- |
| Intermediate SCS (reference) | 1 |  |  |
| Low SCS | 2.56 | 1.19 | 5.50 |
| High SCS | 1.68 | 0.73 | 3.88 |
| ACCI > 2 | 2.08 | 1.21 | 3.59 |
| Pre-operative albumin <35g/l | 2.00 | 1.14 | 3.50 |
| Pre-operative PCI ≤5 (reference) | 1 |  |  |
| Pre-operative PCI 6-14 | 2.18 | 0.99 | 4.79 |
| Pre-operative PCI≥15 | 3.80 | 1.67 | 8.64 |
